# Cervical Foraminal Changes in Patients with Intermittent Arm Radiculopathy Studied with a New MRI-Compatible Compression Device

**DOI:** 10.3390/jcm12206493

**Published:** 2023-10-12

**Authors:** John Hutchins, Hanna Hebelka, Pär-Arne Svensson, Tor Åge Myklebust, Kerstin Lagerstrand, Helena Brisby

**Affiliations:** 1 Institute of Clinical Sciences, Sahlgrenska Academy, University of Gothenburg, SE413 45 Gothenburg, Sweden; 2 Department of Orthopaedics, Sahlgrenska University Hospital, SE413 45 Gothenburg, Sweden; 3 Department of Radiology, Sahlgrenska University Hospital, SE413 45 Gothenburg, Sweden; 4 Department of Registration, Cancer Registry Norway, 0379 Oslo, Norway; 5 Department of Medical Physics and Biomedical Engineering, Sahlgrenska University Hospital, SE413 45 Gothenburg, Sweden

**Keywords:** cervical spine, dynamic MRI, foraminal stenosis, kinetic MRI, spurling test

## Abstract

Diagnosing cervical foraminal stenosis with intermittent arm radiculopathy is challenging due to discrepancies between MRI findings and symptoms. This can be attributed to the fact that MRI images are often obtained in a relaxed supine position. This study aims to evaluate the feasibility of the Dynamic MRI Compression System (DMRICS) and to assess possible changes in cervical foramina, with both quantitative measurements and qualitative grading systems, with MRI during a simulated Spurling test. Ten patients (five women and five men, ages 29–45) with previously confirmed cervical foraminal stenosis underwent MRI scans using DMRICS. MRI images were acquired in both relaxed and provoked states. A radiologist assessed 30 foramina (C4–C7) on the symptomatic side in both patient positions. Quantitative and qualitative measures were performed, including the numeric rating scale (NRS) and the Park and Kim grading systems. The provoked state induced concordant neck and arm pain in 9 of 10 patients. Significant shifts in Park and Kim foraminal gradings were noted: 13 of 27 Park gradings and 9 of 27 Kim gradings escalated post provocation. No quantitative changes were observed. This pilot study indicates that the DMRICS device has the potential to improve diagnostic accuracy for cervical radiculopathy, demonstrating induced cervical foraminal changes during a simulated Spurling test while performing MRI.

## 1. Introduction

The cervical vertebral foramina serve as lateral canals through which nerve roots exit the vertebral column. Located within a motion segment, these foramina are susceptible to degenerative changes over time, potentially resulting in cervical foraminal stenosis [1,2]. Cervical foraminal stenosis with radiculopathy has a prevalence ranging from 121 to 507 per 100,000 individuals [3].

Diagnosing cervical spine conditions is challenging due to discrepancies that often exist between MRI findings and clinical presentations. This discord may stem from the conventional practice of obtaining MRI images while patients are in a relaxed supine position, a state that might not accurately reflect the pathology observed when patients adopt functional positions. Such positions often exacerbate radiculopathy, making dynamic head movements crucial for differential diagnosis and identification of affected nerve roots, as demonstrated by the Spurling test [4]. This diagnostic tool employs cervical spine dynamics and often complements cervical MRI to localize the pain source and identify narrow foramina [5]. A systematic review has shown the Spurling test to possess high specificity (0.89–1.00, 95% confidence interval (CI) 0.59–1.00) and moderate sensitivity (0.38 to 0.97, 95% CI 0.21–0.99) for detecting cervical foraminal stenosis [6].

In the realm of lumbar spine imaging, axial loading of the spine during MRI acquisition has been reported to alter both central and foraminal canal properties. This adjustment enhances diagnostic precision in cases where unloaded MRI do not align with patient symptoms [7,8,9]. Given these findings, questions arise regarding the potential masking of cervical spine pathology due to the relaxed supine position adopted during diagnostic MRI. Visualizing foramina in positions that simulate the Spurling test might be particularly beneficial when multiple foraminal stenoses are present, making it challenging to identify the origin of symptoms.

Prior research has attempted to study cervical foramina using various static head positions, including flexion and extension [10], as well as complex combined movements [11]. Although these studies observed some effects on the foraminal area, their focus was primarily on healthy individuals, and the techniques employed did not allow for dynamic adjustments to head positioning in response to symptom changes or provision of pain exacerbation feedback.

A recent study, utilizing the Dynamic MRI Compression System (DMRICS^®^), demonstrated substantial alterations in cervical foramina in healthy individuals compared with images taken in a relaxed state [12]. The DMRICS^®^ offers a systematic position and force control of the cervical spine within the MRI gantry. It can simulate a Spurling test, which offers the possibility of imaging the spine as symptoms arise and may thereby improve diagnostic accuracy for cervical radiculopathy.

This study aims to explore the feasibility of using DMRICS in patients experiencing intermittent arm radiculopathy, and further, to assess any observed changes in cervical foramina through both quantitative measurements and grading systems during a simulated Spurling test on MRI.

## 2. Materials and Methods

Ten patients that referred to our spine surgery unit between March and May 2022 for suspected cervical foraminal stenosis were consecutively included. The inclusion criteria were intermittent arm radiculopathy with a positive Spurling test, age between 20 and 60 years, and a recently conducted MRI showing one or two ipsilateral stenotic foramina that might explain the symptoms. All patients underwent a new MRI scan in the DIMRICS, first in a relaxed supine position and thereafter with the neck in an extended and lateral flexed position with a slight rotation, including an applied axial load, to simulate the position of a Spurling test [5]. The position and load of the head/neck were slowly applied until a position where the patients experienced radiating arm pain or could not withstand more pressure was reached. Two image acquisitions were obtained, first in the relaxed position and then in the finally reached provoked position. The Neck Disability Index (NDI) and EQ-5D 5L were collected right before the MRI. All participants filled out a numeric rating scale directly before and after the examination, reporting neck and arm pain. Further, the patients answered if the possible change in pain during the examination was concordant with the pain experienced on a daily basis. All included patients signed a written informed consent document before inclusion, and the procedures were performed in accordance with the Declaration of Helsinki and approved by the Regional Ethical Review Board in Gothenburg, Sweden (Dnr [574-18]).

### 2.1. The Compression Device

**Figure 1:**
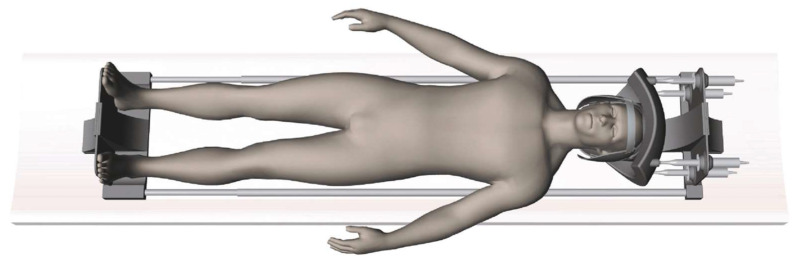
An illustration of a patient in the DMRICS system in a relaxed position.

The Dynamic MRI Compression System, an MRI-compatible compression device, had previously been developed to apply controlled forces to the head and neck during image acquisition (Figure 1). The device utilizes a water hydraulic system with four cylinders connected to a helmet-like structure on the patient’s head, which is linked to an adjustable footplate for varying patient heights. The helmet’s hydraulic cylinders connect to tubes leading to the control room cylinders, which are attached to linear actuators for real-time force control and monitoring. This enables flexion, extension, lateral flexion, rotation, and axial compression adjustments. To reach the Spurling position, force is applied to the posterior cylinders and then to the side that the head is flexed towards. A time-stamped log tracks all actions using custom-developed software. During the compression, the relative forces exerted on each hydraulic cylinder were measured and the data were saved in the log. Instead of calibrating the forces applied in kilograms or pounds, it provides a relative force measurement during compression. The DMRICS has previously only been tested on healthy adults [12].

### 2.2. Image Acquisition and Analysis

Using a 3T MRI scanner (GE 3T Architect Medical Systems, 3000 N Grandview Blvd, Waukesha, WI 53188, USA) and an air coil, T2-weighted (T2w) fast spin echo images were obtained in the oblique sagittal plane with repetition time (TR) 3910 ms, echo time (TE) 102 ms, field of view (FOV) 27 × 27 cm^2^, matrix size 280 × 280, and voxel size 1.0 × 1.0 × 1.0 mm^3^. For the axial plane, TR was 1292 ms, TE 90.85 ms, FOV 26 × 26 cm^2^, matrix 400 × 280 and voxel size 0.6 × 0.9 × 5.0 mm^3^. The latest GE Software SW 29.1 was used (3000 N Grandview Blvd, Waukesha, WI 53188, USA). The images in the oblique sagittal plane were evaluated only on the symptomatic side. The exact same scan protocol was applied in both the relaxed supine position and the subsequent provoked position. The angles for image caption of oblique sagittal and axial scans were chosen to target the foramina in the suspected level(s) as optimally as possible. Multiplanar reconstruction was used only to fine pitch the angles to find the best possible view. Standard protocols for image storage practices were employed throughout the process.

### 2.3. Measurements and Observers

A radiologist (>15 years of experience) assessed the foraminal properties of C4–C7 on the symptomatic side of images both in the relaxed and in the provoked positions. All investigations were coded for blinding purposes. In all included patients, the assessed levels included the clinically suspected stenotic foramina (from the MRI at referral). Two quantitative measures were utilized: the smallest foraminal area on the oblique sagittal images and the foraminal cross distance in the narrowest axial image plane. Additionally, the radiologist employed the Park and Kim grading systems [13,14] to classify foraminal stenosis in both image planes and the Pfirrmann classification system [15] to grade disc degeneration for the discs levels at all examined foramina. The clinic’s standard software, Agfa Enterprise Imaging, was used for all measurements and classifications. Intra- and inter-reliability of the measurements was tested in a previous pilot study of the DMRICS, proving excellent intrarater reliability (>0.98) and good interrater reliability (>0.62) [12].

### 2.4. Statistical Analysis

The Mann–Whitney U-test was used when comparing differences between groups. When comparing differences within groups, the Wilcoxon matched-pairs sign-rank test was used. The Spearman rank correlation was used to estimate the degree of association between quantitative and qualitative measures (Park and Kim). To estimate the effect size for differences in qualitative measures between groups, we estimated ordinal logistic regressions and reported odds ratios with corresponding 95% confidence intervals (CIs). The significance level was set to 5%. The NDI was interpreted with guidance from Vernon et al. [16] that suggests that a score between 0 and 4 represents no disability, 5 and 14 mild disability, 15 and 24 moderate disability, 25 and 34 severe disability, and greater than 35 complete disability. All analyses were performed using SPSS software version 28 and Stata version 17.0.

## 3. Results

### 3.1. Baseline Data

The study population consisted of 10 patients resulting in 30 examined foramina. The baseline data and level of disc degeneration are displayed in Table 1.

### 3.2. Compliance and Patient Experience of the DMRICS Equipment

**Figure 2:**
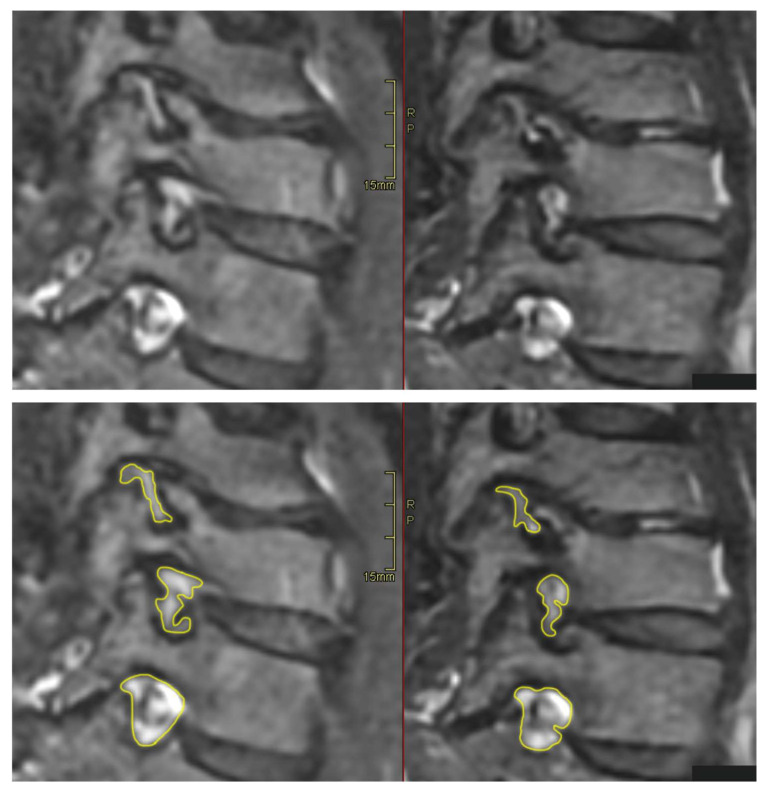
The upper (**left**) image depicts an oblique image in a relaxed position compared with the (**right**) image taken during the Spurling test. The foraminal areas are marked in the lower pair of images.

**Figure 3:**
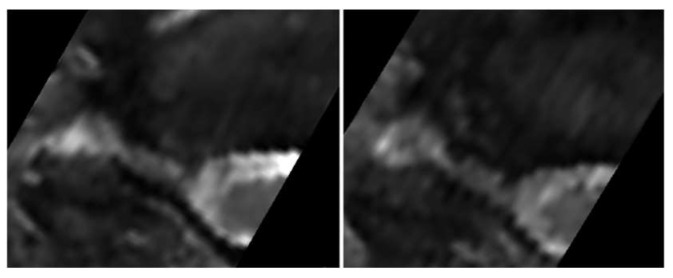
The (**left**) image shows the transverse plane in a relaxed position compared with the (**right**) image taken during the Spurling test.

All patients were positioned without any complaints in the DMRICS apparatus, outside the MRI gantry. The patients were subsequently positioned within the MRI for a relaxed image acquisition. After successfully conducting the simulated Spurling test, immediate imaging was performed, which produced clinically acceptable image quality in 9 out of 10 patients in both relaxed and provoked positions, as shown in Figure 2 and Figure 3.

### 3.3. Applied Force by DMRICS

In 7/9 patients, there was 50% or stronger force applied to the ipsilateral posterior cylinder compared with the contralateral anterior cylinder to achieve a simulated Spurling test.

### 3.4. Changes in NRS Scores in Neck and Arm and If the Pain Was Concordant

During the simulated Spurling test, the neck pain NRS scores increased from 4.6 (standard deviation (SD): 1.84) to 6.8 (SD: 2.25, *p* = 0.011), and the arm pain NRS scores increased from 5.2 (SD: 2.94) to 6.7 (SD: 3.09, *p* = 0.017). A total of 9 out of 10 of the patients reported that the arm pain experienced during the application of forces by the DMRICS was concordant with their ordinary intermittent arm pain symptoms.

### 3.5. Qualitative Grading of Foramina in Relaxed and Provoked Positions

**Figure 4:**
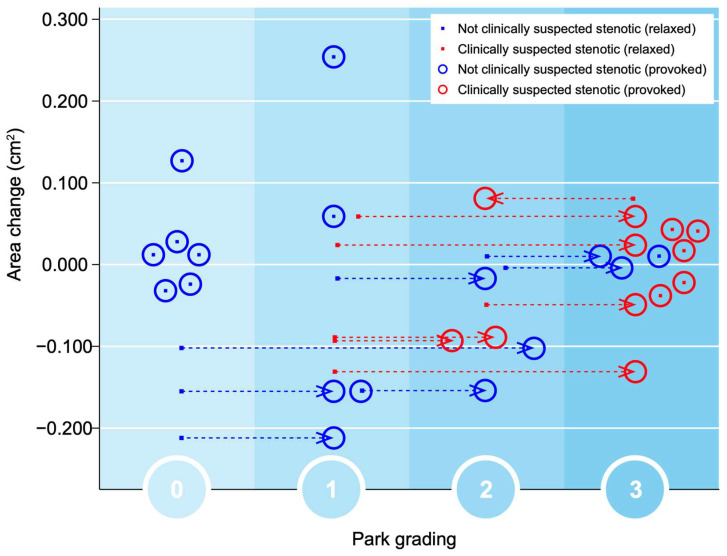
The foraminal area change is demonstrated in relation to the Park classification (0–3). The foramina labeled as clinically suspected stenotic (from the MRI at referral) are marked with a red dot and not suspected are marked with a blue dot. The red circles represent how the suspected foramina were classified after provocation; similarly, the blue circles represent this for the non-suspected foramina.

Significant changes in the gradings of Park and Kim classifications, respectively, after provocation (both *p* = 0.000) were seen. After applying the simulated Spurling test, all initially labeled stenotic foramina were classified as Park grade 2 or 3, with some moving up by 1 grade and others by 2 grades. A total of 13 out of 27 gradings by Park and 9 out of 27 gradings by Kim escalated to a higher grade after provocation. However, there were no significant alterations in the quantitative assessments. In foramina with high Park gradings (2 or 3) in the relaxed images, only minor area changes were observed, depicting that the degenerative segments are less dynamic, as seen in Figure 4.

**Figure 5:**
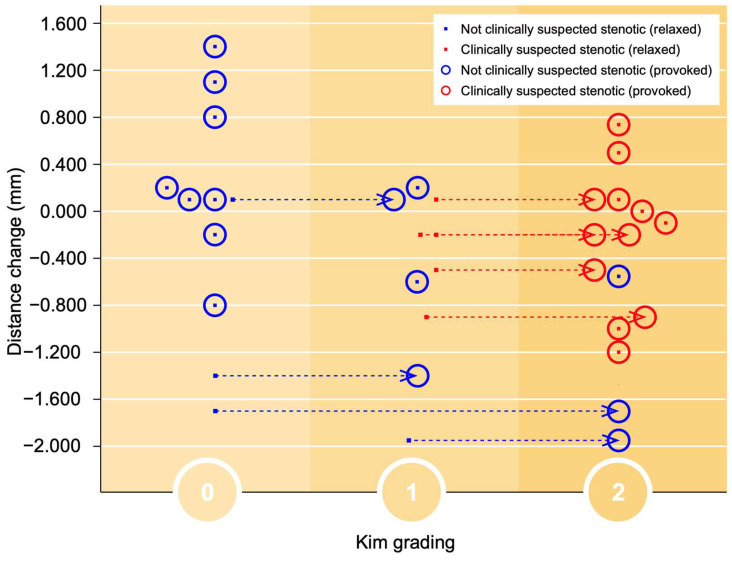
The foraminal cross-distance change is demonstrated in relation to the Kim classification (0–2). The foramina labeled as clinically suspected stenotic (from the MRI at referral) are marked with a red dot and non-stenotic with a blue dot. The red circles represent how the suspected foramina were classified after provocation, similarly the blue circles for the non-suspected foramina.

In accordance with the foraminal changes related to Park gradings during provocation, the foraminal Kim gradings during provocation were all assessed as grade 2, compared with 1 or 2 in the relaxed position, as seen in Figure 5.

### 3.6. Quantitative Measurements of Foramina in Relaxed and Provoked Position

**Figure 6:**
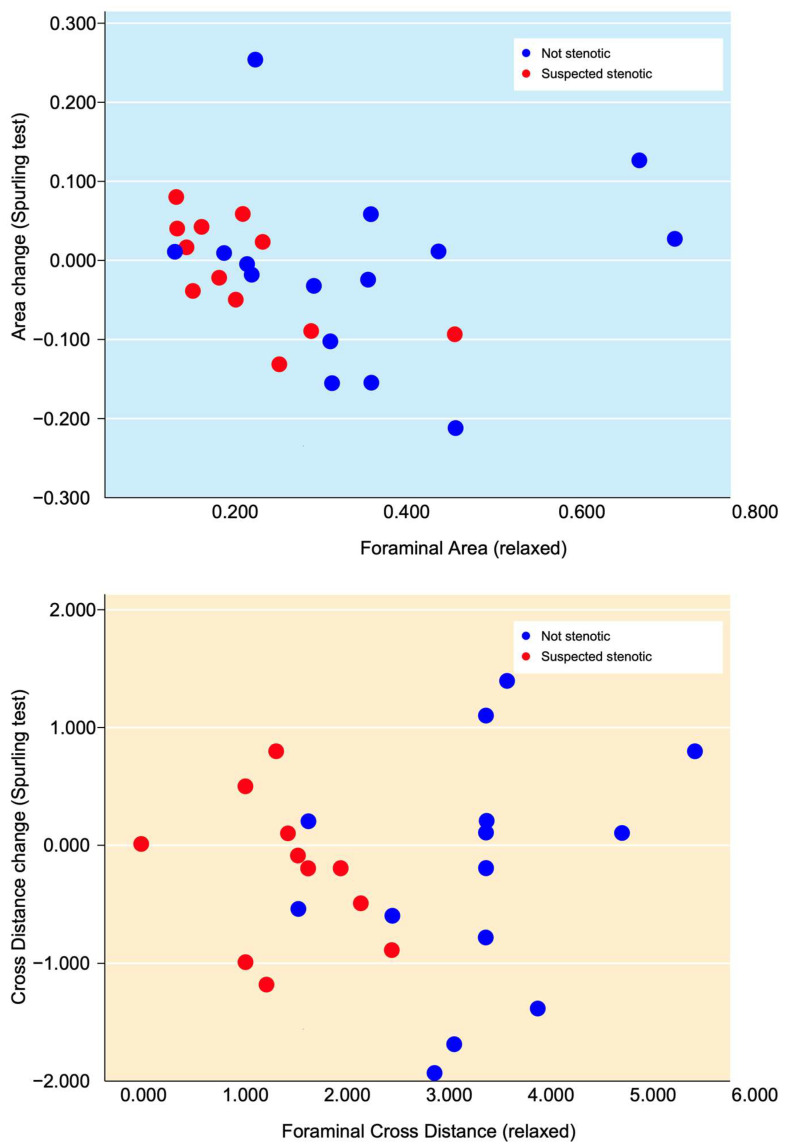
In the upper image, the area changes after the simulated Spurling test are represented on the *Y*-axis and plotted against the relaxed foraminal area on the *x*-axis. In the lower image, the changes in the cross distance on the *y*-axis in relation to the relaxed cross distance can be seen. The foramina clinically suspected to cause the radiculopathy (at referral) are marked as red and the non-clinically suspected foramina as blue.

The changes in all foraminal area and cross-distance measurements after the simulated Spurling test did not show any statistically significant difference, being −0.013 cm^2^ (*p* > 0.503) and −0.21 mm (*p* > 0.260), respectively. Neither were any statistical differences found for area or cross-distance changes in suspected nor for not-suspected foramina when comparing measurements at rest and during provocation. The clinically suspected stenotic foramina (from the MRI at referral) had lower values for both area and cross distance and also small alterations in area size and cross distance after provocation, as seen in Figure 6.

### 3.7. Changes in Foramina in Relation to Disc Degeneration

In the simulated Spurling test, 12/18 foramina adjacent to discs with Pfirrmann grades 2 or 3 exhibited a reduction in the measured area, while 7/9 foramina next to discs with Pfirrmann grades 4 or 5 increased in size. Furthermore, 11/18 foramina neighboring discs with Pfirrmann grades 2 or 3 showed a decrease in the cross distance following the provocation. In contrast, foramina adjacent to discs with Pfirrmann grades 4 or 5 showed mixed results: three foramina increased, five decreased, and one remained unchanged in the cross distance.

### 3.8. Qualitative Grading Systems in Relation to Suspected Levels and Area/Distance Measurements

If the pre-referral MRI indicated potential foraminal stenosis, the likelihood of being categorized in a more advanced Park classification was amplified by a factor of 18.6 (*p* = 0.002). Similarly, the probability of landing in a higher Kim classification increased by a factor of 19.6 (*p* = 0.032).

There was a strong association between the measured area and cross distances in relation to the assigned classification level for the Park (*p* = 0.000) and Kim (*p* = 0.000) systems, during both relaxed and provoked positions.

## 4. Discussion

This pilot study examined the feasibility of the DMRICS in patients with intermittent arm radiculopathy, showing that the device was well tolerated by all patients. The obtained images were of diagnostic quality during provocation except for one patient. The device was able to simulate the Spurling test, resulting in concordant pain in 9/10 of the patients and changes in nerve compromise grading but not quantitative measures of the foramina.

The significant increase in the qualitative gradings of the foramina when comparing MRI before and during the simulated Spurling test indicates that a Spurling test leads to higher Park and Kim grades. This offers not only insights into the variations of the often-employed clinical Spurling test but also suggests a possible clinical utility for dynamic MRI. The method could help in determining the severity of foraminal stenosis and identifying the impacted nerve roots if they are not distinctly discernible in a routine, relaxed MRI, especially when several foramina are narrow on a routine MRI and the symptoms and clinical examination cannot discriminate which nerve root(s) are giving rise to the experienced pain. However, for the foraminal area and cross-distance measures, no significant changes were detected in the present work. The reason for these contradictory findings may be that the foraminal shape shifts with compression and thereby the nerve compression may increase, even if the overall area is not changed or even increases; see Figure 2 and Figure 3. Further, the magnitude of measurement error in such small structures, especially for the axial-cross-distance measurements may also play a role here.

Obtaining MRI of the cervical spine in different positions has been conducted in different settings before [17]. A significant portion of this research has focused on cervical myelopathy, emphasizing the fact that the central canal narrows in extension [18]. Adding axial load to both the cervical spine [19] and lumbar spine [20] implies that clinically valuable information can be revealed with the help of dynamic imaging.

Bartlett and colleagues [21] provided empirical evidence illustrating cervical foraminal changes in images captured during both flexion and extension. Subsequently, a more comprehensive study conducted by Muhle et al. [10] expanded upon these findings. Muhle’s research included not only images taken in flexion and extension but also those captured during rotation, thereby demonstrating foraminal area changes across various positions on the oblique image plane. In this study, the research team employed an MRI-compatible head fixation device, which facilitated the accurate positioning of the head during image acquisition. This device was, however, a fixed construct, placing the head and cervical spine in a static unloaded position, and the included individuals were healthy subjects without any neck or arm pain.

More similar to what was performed in our study, Takasaki and colleagues [11] conducted MRI scans in various static head positions, including during a simulated Spurling test. However, this was also performed on a healthy group of individuals (23 participants, average age of 24.5 years). A reduction in the foraminal area on the ipsilateral side of the executed Spurling test was observed on the oblique sagittal image plane. In our study, wherein we focused on patients experiencing intermittent radiculopathy, averaging 44.5 years in age, we found no such measurable area reduction. Differences in the biomechanical properties of the cervical spine and intervertebral discs under varying degrees of degeneration may be a reason for this, with possible more changes in the foraminal form than the area in patients with more degeneration. This may explain the somewhat conflicting findings between the present study on patients with known degeneration and previous studies on healthy subjects regarding quantitative measurements as being in opposition to individuals with less degenerated higher discs. This was supported by our findings that foramina adjacent to discs with lower Pfirrmann grades (2 or 3) showed a reduction in area and cross distance following the simulated Spurling test, while foramina next to discs with higher Pfirrmann grades (4 or 5) exhibited mixed results. These findings are in accordance with findings in the lumbar spine where degenerative motion segments had a smaller range of motion compared with less degenerated segments [22].

Our study differed conceptually from previous ones in its approach to applying the Spurling test. Rather than keeping patients in a static position, we progressively adjusted their head and cervical position in a manner that mimics the Spurling test as conducted in clinical settings. As we altered the patients’ positions, they were instructed to vocalize their level of discomfort. Upon reaching their discomfort threshold, we halted the provocation, capturing images at that specific position. Out of ten patients, only one produced images during the simulated test that were not of optimal quality due to motion artifacts. Nonetheless, each sequence of images was obtained swiftly, within a time frame of under three minutes, which likely facilitated patient acceptance and cooperation during the process.

In both the initial MRI scans at referral and the new relaxed MRI scans in this study, we found a strong correlation between the severity of foraminal stenosis (quantified through measurements) and the Park and Kim classification systems. These findings align with previous studies [13,14]. Notably, images taken during provocation showed even higher scores in the Park and Kim classifications, reinforcing the reliability of these systems. This suggests the potential clinical utility of imaging in a provoked position for a more accurate diagnosis.

### 4.1. Clinical Relevance and Future Perspective

The DMRICS holds the potential for identifying specific nerve roots that may be causing patients’ symptoms, especially when clinical indications are not clear. This ability is crucial for facilitating targeted treatments, like selective nerve root blocks or surgeries that address a limited number of levels. Furthermore, this study takes into account the patients’ experiences with the DMRICS device, documenting alterations in pain levels before and during the simulated Spurling test. This approach not only provides valuable insights but also underscores the potential clinical utility of the device. To further substantiate the findings of the present study, future research involving larger participant cohorts is imperative. Such studies should aim to validate the efficacy of the DMRICS in distinguishing between nerve roots that signal pain due to compression and those located in narrow foramina without pain signaling among patients suffering from cervical radiculopathy. This future research will play a key role in corroborating the diagnostic accuracy of the DMRICS and its utility in clinical settings for managing cervical radiculopathy.

### 4.2. Strengths and Limitations

The strengths of this study include its novelty, as it utilizes a newly developed MRI-compatible compression device to simulate the Spurling test during MRI acquisition. Furthermore, the study employs a thorough and well-defined methodology, including the use of multiple quantitative and qualitative measures for assessing cervical foramina.

A limitation is that the present study is a pilot study including only a small sample size, with low statistical power. This may have a reduced possibility of detecting small differences in quantitative measurements of the foraminal area and cross distance, especially for the degenerated symptomatic levels. Moreover, the limited resolution of conventional MRI can pose challenges in accurately assessing area and cross-distance measurements due to the substantial influence of partial volume effects. These imperfections in the images may be less consequential when performing qualitative evaluations as opposed to precise quantitative measurements.

## 5. Conclusions

In conclusion, this pilot study demonstrates the potential value of the DMRICS in simulating the Spurling test in a supine position and assessing cervical foraminal changes in patients with intermittent arm radiculopathy. As shown by the present findings, the device may enhance the diagnostic accuracy of cervical radiculopathy by providing additional information on the biomechanical behavior of the cervical spine and foraminal dimensions during provocation. The increase in qualitative gradings of the foramina during compression offers deeper insights into the foraminal changes under provocation. Further research with larger sample sizes and diverse patient populations is warranted to validate the clinical utility of the DMRICS in the diagnosis and management of cervical radiculopathy.

## 6. Patents

The patent of the DMRICS is pending (No. 2251201-6).

## Figures and Tables

**Table 1:** Baseline characteristics.

**Age** mean (range)	44.5 (29–45)
**EQ5D** mean (SD ^1^)	0.6 (0.18)
**NDI** ^2^ mean (SD)	26.6 (10.3)
**NRS** ^3^ **Neck** mean (SD)	4.6 (1.84)
**NRS** ^3^ **Arm** mean (SD)	5.2 (2.95)
	
**Pfirrmann** Grade	Frequency (%)
1	-
2	5 (16.7)
3	15 (50.0)
4	9 (30.0)
5	1 (3.3)

^1^ SD = Standard Deviation, ^2^ NDI = Neck Disability Index, ^3^ NRS = Numerical Rating Scale.

## Data Availability

The measurement data details are available upon request from the corresponding author. The MRI data are not publicly available due to ethical and privacy policy reasons.
